# 9,9′-(Biphenyl-2,2′-di­yl)difluoren-9-ol 4-methyl­pyridine solvate

**DOI:** 10.1107/S1600536810027753

**Published:** 2010-07-21

**Authors:** Jamshid Ashurov, Lidiya Izotova, Aziz Ibragimov, Edwin Weber

**Affiliations:** a Institute of Bioorganic Chemistry, Academy of Sciences of Uzbekistan, H. Abdullaev Str. 83, Tashkent, 100125 Uzbekistan; b National University of Uzbekistan, Faculty of Chemistry, Vuzgorodok, 174, Tashkent, 100174 Uzbekistan; c Institute für Organische Chemie,TU, Bergakademie Freiberg, Leipziger Strasse 29, D-09596 Freiberg/Sachsen, Germany

## Abstract

The title compound, C_38_H_26_O_2_·C_6_H_7_N, crystallized as a host–guest complex from a solvent mixture of 4-methyl­pyridine and acetone. The dihedral angle between the rings in the biphenyl unit is 87.06 (3)°. The methyl­pyridine guest mol­ecules are linked to the host mol­ecules *via* O—H⋯ N hydrogen bonds, forming discrete pairs. The other OH group of the host forms an intra­molecular O—H⋯O hydrogen bond.

## Related literature

For the synthesis of the host compound, see: Weber *et al.* (1993 ▶). For related structures, see: Barbour *et al.* (1993 ▶); Ibragimov *et al.* (2001 ▶); Izotova *et al.* (2008 ▶); Sardone (1996 ▶); Weber (1996 ▶).
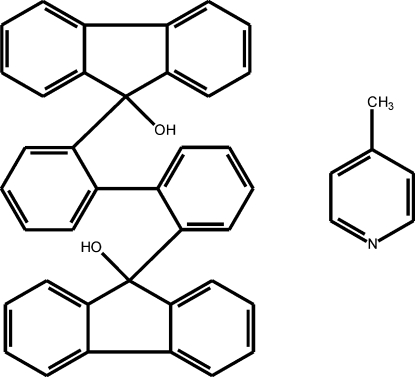


## Experimental

### 

#### Crystal data

C_38_H_26_O_2_·C_6_H_7_N
*M* *_r_* = 607.71
Monoclinic, 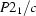

*a* = 15.801 (3) Å
*b* = 15.602 (3) Å
*c* = 14.136 (3) Å
β = 110.19 (3)°
*V* = 3270.8 (11) Å^3^
*Z* = 4
Cu *K*α radiation
μ = 0.58 mm^−1^
*T* = 293 K
0.58 × 0.56 × 0.4 mm

#### Data collection

Oxford Diffraction Xcalibur Ruby diffractometer
Absorption correction: multi-scan (*CrysAlis PRO*; Oxford Diffraction, 2007 ▶) *T*_min_ = 0.749, *T*_max_ = 0.792
46372 measured reflections
6787 independent reflections
5450 reflections with *I* > 2σ(*I*)
*R*_int_ = 0.035

#### Refinement

*R*[*F* ^2^ > 2σ(*F* ^2^)] = 0.041
*wR*(*F* ^2^) = 0.122
*S* = 1.06
6787 reflections
546 parameters
H atoms treated by a mixture of independent and constrained refinement
Δρ_max_ = 0.21 e Å^−3^
Δρ_min_ = −0.18 e Å^−3^

### 

Data collection: *CrysAlis PRO* (Oxford Diffraction, 2007 ▶); cell refinement: *CrysAlis PRO*; data reduction: *CrysAlis PRO*; program(s) used to solve structure: *SHELXS97* (Sheldrick, 2008 ▶); program(s) used to refine structure: *SHELXL97* (Sheldrick, 2008 ▶); molecular graphics: *XP* (Siemens, 1994 ▶) and *Mercury* (Macrae *et al.*, 2006 ▶); software used to prepare material for publication: *SHELXL97*.

## Supplementary Material

Crystal structure: contains datablocks I, global. DOI: 10.1107/S1600536810027753/zl2280sup1.cif

Structure factors: contains datablocks I. DOI: 10.1107/S1600536810027753/zl2280Isup2.hkl

Additional supplementary materials:  crystallographic information; 3D view; checkCIF report

## Figures and Tables

**Table 1:** Hydrogen-bond geometry (Å, °)

*D*—H⋯*A*	*D*—H	H⋯*A*	*D*⋯*A*	*D*—H⋯*A*
O2—H2⋯O1	0.93 (2)	1.90 (2)	2.7943 (15)	161.7 (18)
O1—H1⋯N1	0.91 (2)	1.79 (2)	2.6913 (16)	168.7 (18)

**Fig. 1.**
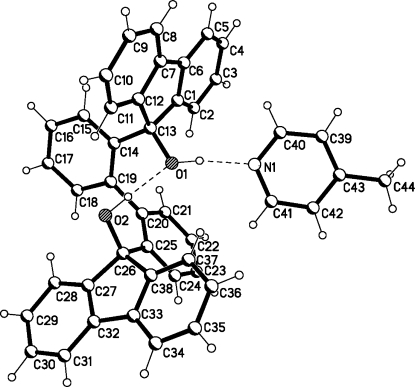
View of the asymmetric unit of the title compound. Hydrogen bonds are indicated as dashed lines.

**Fig. 2.**
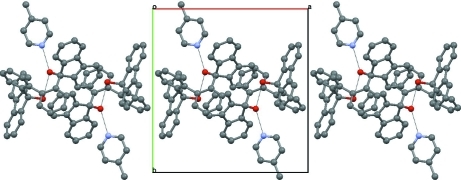
View of the molecular packing along the c axis. Hydrogen atoms omitted for clarity.

## supplementary crystallographic information

### Comment

2,2'-Bis(9-hydroxy-9-fluorenyl) biphenyl is a versatile host compound with the capacity to form inclusion compounds (host–guest complexes) with a variety of guest molecules (about 35 guests reported in the literature, Weber (1996)). In this study we report on the structure of the inclusion compound formed by the host compound with 4-metylpyridine. The host molecule possesses three degrees of freedom for changing its conformation - rotation around the central aryl-aryl single bond and two rotations around the aryl fluorenyl covalent bonds. However, due to the stabilizing effect of the intramolecular hydrogen bond between two hydroxyl groups of the molecule (O2—H···O1, Table 1) the host molecules exhibit a considerable conformational rigidity and are held in a rigid spiral conformation (Fig.1.). In the title compound the hydrogen atom of the second hydroxyl group O1—H points outwards to make a hydrogen bond towards the methylpyridine guest molecule - a structural feature observed in all previously studied structures of the host molecule with polar guests such as acetonitrile, cyclohexanone, n-propylamine (Barbour *et al.*, 1993), acetone (Sardone, 1996; Ibragimov *et al.*, 2001), or ethylacetate (Izotova *et al.*,2008). In the crystals of the title compound the host and guest molecules are connected through intermolecular hydrogen bonds [N···H=1.77 (2) (Å)] which assemble them into 0-dimensional host-guest assemblies. The packing of the assemblies is based on van der Waals interactions (Fig. 2).].

The dihedral angle between the planes of the phenyl rings of the host compound is 87.06 (3)°. The fluorenyl fragments of the host molecule are planar within 0.0382 Å (C1—C13) and 0.0165 Å (C26—C38), respectively. The largest deviations are 0.0695 Å for C13 and 0.0307 Å for C36. The dihedral angle between the planes is 73.73 (4)°.

### Experimental

2,2'-Bis(9-hydroxy-9-fluorenyl) biphenyl (host) was synthesized according to the procedure described by Weber *et al.* (1993). The air stable 4-methylpyridine solvated crystals were obtained by slow evaporation of the host compound from a mixture of acetone and 4-methylpyridine (1:1). The formation of acetone solvated crystals is not observed.

### Refinement

H atoms atoms of the methyl group of 4-methyl pyridine were positioned geometrically, with C—H = 0.96 Å and constrained to ride on their parent atom, with *U*_iso_(H) = 1.5Ueq(C). The remaining H atoms were located in difference syntheses and refined isotropically [O—H = 0.91 (2) and 0.93 (2) Å].

### Crystal data

**Table:**

C_38_H_26_O_2_·C_6_H_7_N	*F*(000) = 1280
*M**_r_* = 607.71	*D*_x_ = 1.234 Mg m^−^^3^
Monoclinic, *P*2_1_/*c*	Cu *K*α radiation, λ = 1.54178 Å
Hall symbol: -P 2ybc	Cell parameters from 19833 reflections
*a* = 15.801 (3) Å	θ = 4.1–75.8°
*b* = 15.602 (3) Å	µ = 0.58 mm^−^^1^
*c* = 14.136 (3) Å	*T* = 293 K
β = 110.19 (3)°	Block, colourless
*V* = 3270.8 (11) Å^3^	0.58 × 0.56 × 0.4 mm
*Z* = 4	

### Data collection

**Table:**

Oxford Diffraction Xcalibur Ruby diffractometer	6787 independent reflections
Radiation source: fine-focus sealed tube	5450 reflections with *I* > 2σ(*I*)
graphite	*R*_int_ = 0.035
Detector resolution: 10.2576 pixels mm^-1^	θ_max_ = 75.8°, θ_min_ = 4.1°
ω scans	*h* = −19→19
Absorption correction: multi-scan (*CrysAlis PRO*; Oxford Diffraction, 2007)	*k* = −18→19
*T*_min_ = 0.749, *T*_max_ = 0.792	*l* = −17→17
46372 measured reflections	

### Refinement

**Table:**

Refinement on *F*^2^	Secondary atom site location: difference Fourier map
Least-squares matrix: full	Hydrogen site location: inferred from neighbouring sites
*R*[*F*^2^ > 2σ(*F*^2^)] = 0.041	H atoms treated by a mixture of independent and constrained refinement
*wR*(*F*^2^) = 0.122	*w* = 1/[σ^2^(*F*_o_^2^) + (0.0748*P*)^2^ + 0.1854*P*] where *P* = (*F*_o_^2^ + 2*F*_c_^2^)/3
*S* = 1.06	(Δ/σ)_max_ = 0.011
6787 reflections	Δρ_max_ = 0.21 e Å^−^^3^
546 parameters	Δρ_min_ = −0.18 e Å^−^^3^
0 restraints	Extinction correction: *SHELXL97* (Sheldrick, 2008), Fc^*^=kFc[1+0.001xFc^2^λ^3^/sin(2θ)]^-1/4^
Primary atom site location: structure-invariant direct methods	Extinction coefficient: 0.0018 (2)

### Special details

**Table:**

Geometry. All e.s.d.'s (except the e.s.d. in the dihedral angle between two l.s. planes) are estimated using the full covariance matrix. The cell e.s.d.'s are taken into account individually in the estimation of e.s.d.'s in distances, angles and torsion angles; correlations between e.s.d.'s in cell parameters are only used when they are defined by crystal symmetry. An approximate (isotropic) treatment of cell e.s.d.'s is used for estimating e.s.d.'s involving l.s. planes.
Refinement. Refinement of *F*^2^ against ALL reflections. The weighted *R*-factor *wR* and goodness of fit *S* are based on *F*^2^, conventional *R*-factors *R* are based on *F*, with *F* set to zero for negative *F*^2^. The threshold expression of *F*^2^ > σ(*F*^2^) is used only for calculating *R*-factors(gt) *etc*. and is not relevant to the choice of reflections for refinement. *R*-factors based on *F*^2^ are statistically about twice as large as those based on *F*, and *R*- factors based on ALL data will be even larger.

### Fractional atomic coordinates and isotropic or equivalent isotropic displacement parameters (Å^2^)

**Table:**

	*x*	*y*	*z*	*U*_iso_*/*U*_eq_	
N1	0.28630 (10)	0.22388 (8)	0.23144 (12)	0.0762 (4)	
C39	0.30568 (13)	0.07376 (12)	0.24668 (15)	0.0794 (5)	
C40	0.33566 (13)	0.15436 (12)	0.23830 (18)	0.0865 (5)	
C41	0.20339 (12)	0.21306 (10)	0.23211 (14)	0.0705 (4)	
C42	0.16876 (11)	0.13410 (10)	0.24206 (12)	0.0666 (4)	
C43	0.22017 (12)	0.06195 (9)	0.24911 (10)	0.0632 (4)	
C44	0.18342 (18)	−0.02567 (12)	0.25497 (16)	0.1027 (7)	
H44C	0.1305	−0.0208	0.2733	0.154*	
H44A	0.2281	−0.0590	0.3048	0.154*	
H44B	0.1681	−0.0533	0.1906	0.154*	
H39	0.3454 (15)	0.0278 (14)	0.2504 (16)	0.102 (7)*	
H40	0.3967 (17)	0.1637 (15)	0.2386 (18)	0.111 (7)*	
H41	0.1658 (13)	0.2628 (13)	0.2245 (14)	0.086 (6)*	
H42	0.1102 (14)	0.1315 (12)	0.2448 (15)	0.088 (6)*	
O1	0.33690 (6)	0.38807 (6)	0.22672 (7)	0.0492 (2)	
O2	0.29222 (6)	0.55099 (7)	0.27689 (7)	0.0571 (2)	
C1	0.42742 (8)	0.33134 (7)	0.12905 (10)	0.0470 (3)	
C2	0.37556 (10)	0.29484 (9)	0.03880 (11)	0.0586 (3)	
C3	0.41119 (13)	0.22588 (10)	0.00192 (13)	0.0696 (4)	
C4	0.49610 (13)	0.19436 (9)	0.05510 (14)	0.0723 (4)	
C5	0.54802 (11)	0.23036 (9)	0.14612 (14)	0.0656 (4)	
C6	0.51351 (9)	0.29984 (8)	0.18251 (10)	0.0520 (3)	
C7	0.55424 (8)	0.35289 (8)	0.27283 (11)	0.0529 (3)	
C8	0.63650 (10)	0.34711 (11)	0.35155 (14)	0.0703 (4)	
C9	0.65709 (11)	0.40714 (13)	0.42803 (14)	0.0775 (5)	
C10	0.59787 (11)	0.47218 (11)	0.42739 (13)	0.0705 (4)	
C11	0.51546 (10)	0.47859 (10)	0.34950 (11)	0.0586 (3)	
C12	0.49418 (8)	0.41807 (8)	0.27288 (9)	0.0478 (3)	
C13	0.40518 (7)	0.40751 (7)	0.18496 (9)	0.0433 (3)	
C14	0.37663 (8)	0.48741 (7)	0.11860 (9)	0.0436 (2)	
C15	0.44456 (10)	0.53162 (9)	0.09671 (12)	0.0574 (3)	
C16	0.42725 (11)	0.60509 (10)	0.03888 (13)	0.0680 (4)	
C17	0.34103 (12)	0.63647 (10)	0.00125 (13)	0.0677 (4)	
C18	0.27204 (10)	0.59256 (9)	0.01963 (11)	0.0572 (3)	
C19	0.28788 (8)	0.51801 (7)	0.07821 (8)	0.0443 (3)	
C20	0.20356 (7)	0.47327 (7)	0.07992 (8)	0.0424 (2)	
C21	0.16213 (9)	0.42064 (9)	−0.00338 (9)	0.0527 (3)	
C22	0.08037 (9)	0.38101 (9)	−0.01885 (10)	0.0581 (3)	
C23	0.03635 (9)	0.39469 (9)	0.04847 (10)	0.0551 (3)	
C24	0.07593 (8)	0.44634 (8)	0.13146 (9)	0.0486 (3)	
C25	0.15942 (7)	0.48553 (7)	0.14956 (8)	0.0422 (2)	
C26	0.19745 (8)	0.53939 (8)	0.24648 (9)	0.0486 (3)	
C27	0.15271 (9)	0.62719 (9)	0.23645 (11)	0.0596 (3)	
C28	0.15284 (12)	0.69314 (10)	0.17149 (15)	0.0752 (4)	
C29	0.10562 (16)	0.76798 (12)	0.1749 (2)	0.1026 (7)	
C30	0.06147 (16)	0.77686 (16)	0.2424 (3)	0.1200 (10)	
C31	0.06224 (13)	0.71165 (17)	0.3087 (2)	0.1020 (8)	
C32	0.10751 (9)	0.63569 (12)	0.30554 (13)	0.0720 (5)	
C33	0.11990 (9)	0.55660 (13)	0.36463 (11)	0.0717 (5)	
C34	0.09016 (13)	0.5321 (2)	0.44281 (16)	0.1002 (8)	
C35	0.11436 (15)	0.4533 (3)	0.48663 (15)	0.1152 (10)	
C36	0.16715 (14)	0.3978 (2)	0.45519 (14)	0.0985 (7)	
C37	0.19693 (11)	0.42109 (14)	0.37662 (11)	0.0729 (4)	
C38	0.17243 (8)	0.49993 (10)	0.33219 (10)	0.0577 (3)	
H1	0.3237 (12)	0.3311 (13)	0.2227 (14)	0.081 (5)*	
H2	0.3139 (13)	0.4960 (13)	0.2749 (15)	0.085 (6)*	
H2A	0.3128 (11)	0.3175 (10)	0.0000 (12)	0.065 (4)*	
H3	0.3744 (13)	0.1986 (12)	−0.0629 (15)	0.084 (5)*	
H4	0.5219 (13)	0.1475 (12)	0.0259 (14)	0.081 (5)*	
H5	0.6077 (13)	0.2082 (11)	0.1823 (13)	0.077 (5)*	
H8	0.6729 (12)	0.3022 (12)	0.3498 (13)	0.074 (5)*	
H9	0.7146 (15)	0.4039 (12)	0.4813 (16)	0.089 (6)*	
H10	0.6156 (13)	0.5164 (12)	0.4842 (15)	0.082 (5)*	
H11	0.4705 (12)	0.5253 (11)	0.3478 (13)	0.071 (5)*	
H15	0.5066 (12)	0.5097 (10)	0.1242 (12)	0.066 (4)*	
H16	0.4775 (13)	0.6340 (12)	0.0264 (15)	0.086 (5)*	
H17	0.3270 (12)	0.6881 (12)	−0.0403 (14)	0.077 (5)*	
H18	0.2106 (12)	0.6142 (10)	−0.0075 (13)	0.068 (4)*	
H21	0.1946 (11)	0.4155 (10)	−0.0559 (12)	0.067 (4)*	
H22	0.0537 (12)	0.3458 (11)	−0.0780 (14)	0.074 (5)*	
H23	−0.0239 (12)	0.3697 (10)	0.0375 (12)	0.068 (4)*	
H24	0.0443 (10)	0.4565 (10)	0.1799 (12)	0.061 (4)*	
H28	0.1918 (13)	0.6868 (12)	0.1288 (14)	0.084 (5)*	
H29	0.1079 (17)	0.8148 (17)	0.1337 (19)	0.117 (8)*	
H30	0.0265 (19)	0.8288 (19)	0.249 (2)	0.145 (10)*	
H31	0.0313 (17)	0.7197 (16)	0.3573 (19)	0.117 (8)*	
H34	0.0531 (17)	0.5695 (15)	0.4681 (19)	0.119 (8)*	
H35	0.0927 (16)	0.4365 (16)	0.5376 (19)	0.116 (7)*	
H36	0.1851 (18)	0.3430 (17)	0.488 (2)	0.125 (9)*	
H37	0.2321 (14)	0.3819 (12)	0.3531 (14)	0.083 (6)*	

### Atomic displacement parameters (Å^2^)

**Table:**

	*U*^11^	*U*^22^	*U*^33^	*U*^12^	*U*^13^	*U*^23^
N1	0.0829 (9)	0.0576 (7)	0.1004 (10)	−0.0149 (6)	0.0472 (8)	−0.0029 (6)
C39	0.0788 (11)	0.0619 (9)	0.0957 (12)	0.0086 (8)	0.0279 (9)	−0.0065 (8)
C40	0.0700 (11)	0.0746 (11)	0.1267 (16)	−0.0055 (8)	0.0491 (11)	−0.0080 (10)
C41	0.0745 (10)	0.0558 (8)	0.0872 (11)	0.0020 (7)	0.0356 (8)	−0.0012 (7)
C42	0.0636 (9)	0.0695 (9)	0.0730 (9)	−0.0106 (7)	0.0316 (7)	−0.0043 (7)
C43	0.0851 (10)	0.0523 (7)	0.0506 (7)	−0.0121 (7)	0.0213 (7)	−0.0021 (5)
C44	0.152 (2)	0.0651 (11)	0.0863 (13)	−0.0351 (12)	0.0351 (13)	0.0005 (9)
O1	0.0466 (4)	0.0488 (5)	0.0612 (5)	−0.0032 (3)	0.0300 (4)	0.0030 (4)
O2	0.0365 (4)	0.0776 (6)	0.0583 (5)	−0.0087 (4)	0.0179 (4)	−0.0200 (4)
C1	0.0486 (6)	0.0417 (6)	0.0587 (7)	−0.0047 (5)	0.0287 (5)	−0.0013 (5)
C2	0.0632 (8)	0.0534 (7)	0.0641 (8)	−0.0096 (6)	0.0283 (7)	−0.0083 (6)
C3	0.0897 (11)	0.0555 (8)	0.0748 (10)	−0.0151 (7)	0.0426 (9)	−0.0158 (7)
C4	0.0960 (12)	0.0450 (7)	0.0989 (12)	−0.0021 (7)	0.0631 (10)	−0.0074 (7)
C5	0.0674 (9)	0.0492 (7)	0.0948 (11)	0.0072 (6)	0.0467 (8)	0.0058 (7)
C6	0.0520 (7)	0.0442 (6)	0.0689 (8)	−0.0002 (5)	0.0327 (6)	0.0043 (5)
C7	0.0443 (6)	0.0528 (7)	0.0646 (7)	−0.0018 (5)	0.0226 (6)	0.0067 (5)
C8	0.0490 (7)	0.0720 (10)	0.0857 (11)	0.0070 (7)	0.0179 (7)	0.0146 (8)
C9	0.0529 (8)	0.0949 (12)	0.0711 (10)	−0.0132 (8)	0.0038 (7)	0.0076 (9)
C10	0.0604 (9)	0.0814 (10)	0.0632 (9)	−0.0163 (8)	0.0131 (7)	−0.0065 (7)
C11	0.0545 (7)	0.0617 (8)	0.0609 (8)	−0.0108 (6)	0.0215 (6)	−0.0089 (6)
C12	0.0422 (6)	0.0496 (6)	0.0543 (7)	−0.0048 (5)	0.0201 (5)	0.0019 (5)
C13	0.0397 (5)	0.0442 (6)	0.0509 (6)	−0.0047 (4)	0.0221 (5)	−0.0029 (4)
C14	0.0439 (6)	0.0438 (6)	0.0491 (6)	−0.0048 (4)	0.0238 (5)	−0.0037 (4)
C15	0.0505 (7)	0.0581 (7)	0.0732 (8)	−0.0066 (6)	0.0336 (7)	0.0025 (6)
C16	0.0701 (9)	0.0628 (8)	0.0861 (10)	−0.0128 (7)	0.0463 (8)	0.0092 (7)
C17	0.0797 (10)	0.0557 (8)	0.0779 (10)	−0.0004 (7)	0.0401 (8)	0.0165 (7)
C18	0.0587 (8)	0.0564 (7)	0.0606 (7)	0.0034 (6)	0.0259 (6)	0.0079 (6)
C19	0.0470 (6)	0.0468 (6)	0.0443 (6)	−0.0040 (5)	0.0224 (5)	−0.0027 (4)
C20	0.0390 (5)	0.0466 (6)	0.0417 (5)	−0.0002 (4)	0.0140 (4)	0.0006 (4)
C21	0.0520 (7)	0.0630 (7)	0.0434 (6)	−0.0029 (6)	0.0168 (5)	−0.0053 (5)
C22	0.0552 (7)	0.0622 (8)	0.0497 (7)	−0.0098 (6)	0.0088 (6)	−0.0111 (6)
C23	0.0429 (6)	0.0607 (7)	0.0562 (7)	−0.0123 (5)	0.0100 (5)	−0.0006 (6)
C24	0.0400 (6)	0.0573 (7)	0.0493 (6)	−0.0049 (5)	0.0164 (5)	−0.0005 (5)
C25	0.0375 (5)	0.0457 (6)	0.0433 (6)	−0.0014 (4)	0.0140 (4)	−0.0015 (4)
C26	0.0366 (5)	0.0616 (7)	0.0503 (6)	−0.0067 (5)	0.0182 (5)	−0.0129 (5)
C27	0.0420 (6)	0.0619 (8)	0.0728 (9)	−0.0088 (5)	0.0174 (6)	−0.0258 (7)
C28	0.0680 (9)	0.0530 (8)	0.1008 (12)	−0.0131 (7)	0.0244 (9)	−0.0193 (8)
C29	0.0922 (14)	0.0532 (10)	0.149 (2)	−0.0084 (9)	0.0251 (14)	−0.0250 (12)
C30	0.0833 (14)	0.0724 (14)	0.198 (3)	0.0004 (11)	0.0401 (16)	−0.0593 (17)
C31	0.0645 (10)	0.1012 (16)	0.146 (2)	−0.0073 (10)	0.0435 (12)	−0.0675 (15)
C32	0.0436 (7)	0.0881 (11)	0.0850 (10)	−0.0084 (7)	0.0233 (7)	−0.0417 (9)
C33	0.0408 (6)	0.1188 (14)	0.0589 (8)	−0.0129 (7)	0.0216 (6)	−0.0340 (8)
C34	0.0558 (9)	0.189 (3)	0.0647 (11)	−0.0137 (12)	0.0324 (8)	−0.0342 (14)
C35	0.0709 (12)	0.229 (3)	0.0525 (10)	−0.0207 (16)	0.0295 (9)	0.0039 (15)
C36	0.0721 (11)	0.162 (2)	0.0577 (10)	−0.0131 (13)	0.0174 (8)	0.0232 (12)
C37	0.0598 (8)	0.1069 (13)	0.0501 (7)	−0.0048 (9)	0.0163 (7)	0.0044 (8)
C38	0.0412 (6)	0.0888 (10)	0.0435 (6)	−0.0095 (6)	0.0152 (5)	−0.0142 (6)

### Geometric parameters (Å, °)

**Table:**

N1—C40	1.320 (2)	C15—C16	1.379 (2)
N1—C41	1.324 (2)	C15—H15	0.984 (17)
C39—C40	1.363 (3)	C16—C17	1.371 (2)
C39—C43	1.376 (3)	C16—H16	0.98 (2)
C39—H39	0.94 (2)	C17—C18	1.386 (2)
C40—H40	0.97 (2)	C17—H17	0.976 (18)
C41—C42	1.375 (2)	C18—C19	1.3994 (18)
C41—H41	0.96 (2)	C18—H18	0.973 (18)
C42—C43	1.372 (2)	C19—C20	1.5117 (15)
C42—H42	0.94 (2)	C20—C21	1.3991 (17)
C43—C44	1.499 (2)	C20—C25	1.4022 (16)
C44—H44C	0.9600	C21—C22	1.3799 (19)
C44—H44A	0.9600	C21—H21	1.041 (17)
C44—H44B	0.9600	C22—C23	1.376 (2)
O1—C13	1.4298 (13)	C22—H22	0.967 (18)
O1—H1	0.91 (2)	C23—C24	1.3820 (18)
O2—C26	1.4197 (14)	C23—H23	0.990 (17)
O2—H2	0.93 (2)	C24—C25	1.3952 (16)
C1—C2	1.3790 (19)	C24—H24	0.991 (16)
C1—C6	1.3976 (18)	C25—C26	1.5414 (16)
C1—C13	1.5338 (16)	C26—C27	1.5256 (19)
C2—C3	1.396 (2)	C26—C38	1.5278 (18)
C2—H2A	1.016 (16)	C27—C28	1.380 (2)
C3—C4	1.382 (3)	C27—C32	1.401 (2)
C3—H3	0.995 (19)	C28—C29	1.395 (3)
C4—C5	1.384 (3)	C28—H28	1.01 (2)
C4—H4	0.994 (19)	C29—C30	1.370 (4)
C5—C6	1.3895 (19)	C29—H29	0.94 (3)
C5—H5	0.969 (18)	C30—C31	1.380 (4)
C6—C7	1.470 (2)	C30—H30	1.00 (3)
C7—C12	1.3910 (18)	C31—C32	1.393 (3)
C7—C8	1.392 (2)	C31—H31	0.98 (3)
C8—C9	1.382 (3)	C32—C33	1.465 (3)
C8—H8	0.912 (18)	C33—C38	1.394 (2)
C9—C10	1.378 (3)	C33—C34	1.395 (3)
C9—H9	0.96 (2)	C34—C35	1.369 (4)
C10—C11	1.389 (2)	C34—H34	0.98 (3)
C10—H10	1.022 (19)	C35—C36	1.379 (4)
C11—C12	1.3879 (19)	C35—H35	0.94 (3)
C11—H11	1.013 (17)	C36—C37	1.396 (2)
C12—C13	1.5306 (18)	C36—H36	0.97 (3)
C13—C14	1.5313 (16)	C37—C38	1.375 (2)
C14—C15	1.3976 (16)	C37—H37	0.96 (2)
C14—C19	1.4028 (17)		
			
C40—N1—C41	117.11 (14)	C17—C16—C15	119.61 (13)
C40—C39—C43	119.96 (16)	C17—C16—H16	121.6 (11)
C40—C39—H39	117.5 (13)	C15—C16—H16	118.8 (11)
C43—C39—H39	122.6 (13)	C16—C17—C18	119.44 (13)
N1—C40—C39	123.38 (17)	C16—C17—H17	121.1 (11)
N1—C40—H40	115.8 (14)	C18—C17—H17	119.4 (11)
C39—C40—H40	120.8 (14)	C17—C18—C19	122.03 (14)
N1—C41—C42	123.00 (16)	C17—C18—H18	119.8 (10)
N1—C41—H41	118.2 (12)	C19—C18—H18	118.2 (10)
C42—C41—H41	118.8 (12)	C18—C19—C14	118.24 (11)
C43—C42—C41	119.75 (15)	C18—C19—C20	114.50 (11)
C43—C42—H42	121.9 (12)	C14—C19—C20	126.81 (10)
C41—C42—H42	118.4 (12)	C21—C20—C25	117.99 (10)
C42—C43—C39	116.77 (14)	C21—C20—C19	114.61 (10)
C42—C43—C44	121.44 (17)	C25—C20—C19	127.09 (10)
C39—C43—C44	121.75 (17)	C22—C21—C20	122.47 (12)
C43—C44—H44C	109.5	C22—C21—H21	121.2 (9)
C43—C44—H44A	109.5	C20—C21—H21	116.3 (9)
H44C—C44—H44A	109.5	C23—C22—C21	119.35 (12)
C43—C44—H44B	109.5	C23—C22—H22	120.9 (10)
H44C—C44—H44B	109.5	C21—C22—H22	119.7 (11)
H44A—C44—H44B	109.5	C22—C23—C24	119.28 (12)
C13—O1—H1	111.7 (12)	C22—C23—H23	121.3 (10)
C26—O2—H2	103.6 (12)	C24—C23—H23	119.4 (10)
C2—C1—C6	120.77 (12)	C23—C24—C25	122.23 (12)
C2—C1—C13	128.91 (12)	C23—C24—H24	119.5 (9)
C6—C1—C13	110.32 (11)	C25—C24—H24	118.3 (9)
C1—C2—C3	118.44 (15)	C24—C25—C20	118.66 (10)
C1—C2—H2A	120.9 (9)	C24—C25—C26	117.11 (10)
C3—C2—H2A	120.7 (9)	C20—C25—C26	124.23 (10)
C4—C3—C2	120.79 (15)	O2—C26—C27	108.68 (10)
C4—C3—H3	119.8 (11)	O2—C26—C38	110.24 (10)
C2—C3—H3	119.4 (11)	C27—C26—C38	101.36 (11)
C3—C4—C5	120.92 (14)	O2—C26—C25	112.68 (9)
C3—C4—H4	119.7 (11)	C27—C26—C25	112.39 (10)
C5—C4—H4	119.2 (11)	C38—C26—C25	110.92 (10)
C4—C5—C6	118.58 (15)	C28—C27—C32	120.77 (15)
C4—C5—H5	120.2 (11)	C28—C27—C26	128.64 (14)
C6—C5—H5	121.2 (11)	C32—C27—C26	110.59 (14)
C5—C6—C1	120.48 (14)	C27—C28—C29	118.5 (2)
C5—C6—C7	130.59 (13)	C27—C28—H28	117.9 (11)
C1—C6—C7	108.91 (11)	C29—C28—H28	123.3 (11)
C12—C7—C8	119.73 (14)	C30—C29—C28	121.0 (3)
C12—C7—C6	108.53 (11)	C30—C29—H29	119.3 (16)
C8—C7—C6	131.74 (14)	C28—C29—H29	119.5 (17)
C9—C8—C7	118.96 (16)	C29—C30—C31	120.7 (2)
C9—C8—H8	124.2 (11)	C29—C30—H30	125.0 (18)
C7—C8—H8	116.8 (11)	C31—C30—H30	114.2 (18)
C10—C9—C8	121.20 (15)	C30—C31—C32	119.3 (2)
C10—C9—H9	120.1 (12)	C30—C31—H31	119.5 (15)
C8—C9—H9	118.7 (12)	C32—C31—H31	121.2 (15)
C9—C10—C11	120.46 (15)	C31—C32—C27	119.6 (2)
C9—C10—H10	119.4 (11)	C31—C32—C33	131.87 (19)
C11—C10—H10	120.1 (11)	C27—C32—C33	108.53 (13)
C12—C11—C10	118.56 (14)	C38—C33—C34	119.2 (2)
C12—C11—H11	119.5 (10)	C38—C33—C32	108.91 (13)
C10—C11—H11	122.0 (10)	C34—C33—C32	131.87 (19)
C11—C12—C7	121.08 (12)	C35—C34—C33	119.1 (2)
C11—C12—C13	127.88 (12)	C35—C34—H34	118.7 (15)
C7—C12—C13	110.90 (11)	C33—C34—H34	122.2 (15)
O1—C13—C14	108.72 (9)	C34—C35—C36	121.6 (2)
O1—C13—C12	107.48 (9)	C34—C35—H35	118.8 (16)
C14—C13—C12	113.81 (9)	C36—C35—H35	119.6 (16)
O1—C13—C1	112.84 (9)	C35—C36—C37	120.0 (2)
C14—C13—C1	112.74 (9)	C35—C36—H36	120.9 (17)
C12—C13—C1	101.07 (9)	C37—C36—H36	119.1 (17)
C15—C14—C19	118.57 (11)	C38—C37—C36	118.5 (2)
C15—C14—C13	116.81 (11)	C38—C37—H37	121.2 (12)
C19—C14—C13	124.62 (10)	C36—C37—H37	120.2 (12)
C16—C15—C14	122.08 (14)	C37—C38—C33	121.53 (15)
C16—C15—H15	119.4 (9)	C37—C38—C26	127.86 (13)
C14—C15—H15	118.5 (10)	C33—C38—C26	110.60 (14)
			
C41—N1—C40—C39	−0.5 (3)	C15—C14—C19—C20	170.49 (11)
C43—C39—C40—N1	−0.5 (3)	C13—C14—C19—C20	−8.82 (18)
C40—N1—C41—C42	1.7 (3)	C18—C19—C20—C21	81.73 (14)
N1—C41—C42—C43	−1.8 (3)	C14—C19—C20—C21	−90.39 (15)
C41—C42—C43—C39	0.6 (2)	C18—C19—C20—C25	−91.68 (15)
C41—C42—C43—C44	−176.98 (16)	C14—C19—C20—C25	96.20 (15)
C40—C39—C43—C42	0.4 (3)	C25—C20—C21—C22	−0.01 (19)
C40—C39—C43—C44	178.04 (19)	C19—C20—C21—C22	−174.06 (12)
C6—C1—C2—C3	−0.20 (19)	C20—C21—C22—C23	1.4 (2)
C13—C1—C2—C3	179.11 (12)	C21—C22—C23—C24	−1.4 (2)
C1—C2—C3—C4	0.6 (2)	C22—C23—C24—C25	0.1 (2)
C2—C3—C4—C5	−0.1 (2)	C23—C24—C25—C20	1.29 (18)
C3—C4—C5—C6	−0.9 (2)	C23—C24—C25—C26	−178.23 (12)
C4—C5—C6—C1	1.31 (19)	C21—C20—C25—C24	−1.31 (17)
C4—C5—C6—C7	−176.71 (13)	C19—C20—C25—C24	171.90 (11)
C2—C1—C6—C5	−0.77 (18)	C21—C20—C25—C26	178.17 (11)
C13—C1—C6—C5	179.79 (11)	C19—C20—C25—C26	−8.61 (18)
C2—C1—C6—C7	177.64 (11)	C24—C25—C26—O2	159.13 (11)
C13—C1—C6—C7	−1.79 (13)	C20—C25—C26—O2	−20.36 (17)
C5—C6—C7—C12	176.47 (13)	C24—C25—C26—C27	−77.67 (14)
C1—C6—C7—C12	−1.72 (14)	C20—C25—C26—C27	102.84 (13)
C5—C6—C7—C8	−4.2 (2)	C24—C25—C26—C38	35.00 (15)
C1—C6—C7—C8	177.62 (15)	C20—C25—C26—C38	−144.49 (11)
C12—C7—C8—C9	−0.6 (2)	O2—C26—C27—C28	62.88 (17)
C6—C7—C8—C9	−179.85 (14)	C38—C26—C27—C28	179.01 (14)
C7—C8—C9—C10	−0.1 (3)	C25—C26—C27—C28	−62.53 (17)
C8—C9—C10—C11	0.3 (3)	O2—C26—C27—C32	−116.98 (12)
C9—C10—C11—C12	0.2 (2)	C38—C26—C27—C32	−0.86 (13)
C10—C11—C12—C7	−0.9 (2)	C25—C26—C27—C32	117.61 (12)
C10—C11—C12—C13	174.32 (13)	C32—C27—C28—C29	−1.1 (2)
C8—C7—C12—C11	1.06 (19)	C26—C27—C28—C29	179.03 (14)
C6—C7—C12—C11	−179.51 (11)	C27—C28—C29—C30	1.3 (3)
C8—C7—C12—C13	−174.87 (12)	C28—C29—C30—C31	−0.3 (3)
C6—C7—C12—C13	4.57 (13)	C29—C30—C31—C32	−0.9 (3)
C11—C12—C13—O1	−62.44 (15)	C30—C31—C32—C27	1.0 (3)
C7—C12—C13—O1	113.13 (11)	C30—C31—C32—C33	−179.83 (18)
C11—C12—C13—C14	58.00 (16)	C28—C27—C32—C31	0.0 (2)
C7—C12—C13—C14	−126.43 (11)	C26—C27—C32—C31	179.84 (13)
C11—C12—C13—C1	179.12 (12)	C28—C27—C32—C33	−179.35 (13)
C7—C12—C13—C1	−5.31 (12)	C26—C27—C32—C33	0.52 (15)
C2—C1—C13—O1	70.32 (16)	C31—C32—C33—C38	−179.12 (16)
C6—C1—C13—O1	−110.30 (11)	C27—C32—C33—C38	0.08 (16)
C2—C1—C13—C14	−53.32 (16)	C31—C32—C33—C34	0.1 (3)
C6—C1—C13—C14	126.06 (11)	C27—C32—C33—C34	179.33 (16)
C2—C1—C13—C12	−175.19 (12)	C38—C33—C34—C35	0.9 (3)
C6—C1—C13—C12	4.19 (12)	C32—C33—C34—C35	−178.31 (17)
O1—C13—C14—C15	160.77 (11)	C33—C34—C35—C36	−0.2 (3)
C12—C13—C14—C15	41.03 (15)	C34—C35—C36—C37	−0.3 (3)
C1—C13—C14—C15	−73.33 (14)	C35—C36—C37—C38	0.0 (3)
O1—C13—C14—C19	−19.91 (15)	C36—C37—C38—C33	0.7 (2)
C12—C13—C14—C19	−139.65 (11)	C36—C37—C38—C26	179.36 (14)
C1—C13—C14—C19	105.99 (13)	C34—C33—C38—C37	−1.2 (2)
C19—C14—C15—C16	1.5 (2)	C32—C33—C38—C37	178.19 (13)
C13—C14—C15—C16	−179.09 (13)	C34—C33—C38—C26	179.98 (13)
C14—C15—C16—C17	0.0 (2)	C32—C33—C38—C26	−0.66 (15)
C15—C16—C17—C18	−1.7 (3)	O2—C26—C38—C37	−62.87 (17)
C16—C17—C18—C19	1.9 (2)	C27—C26—C38—C37	−177.84 (14)
C17—C18—C19—C14	−0.3 (2)	C25—C26—C38—C37	62.64 (17)
C17—C18—C19—C20	−173.15 (13)	O2—C26—C38—C33	115.88 (12)
C15—C14—C19—C18	−1.37 (17)	C27—C26—C38—C33	0.91 (13)
C13—C14—C19—C18	179.32 (11)	C25—C26—C38—C33	−118.61 (12)

### Hydrogen-bond geometry (Å, °)

**Table:**

*D*—H···*A*	*D*—H	H···*A*	*D*···*A*	*D*—H···*A*
O2—H2···O1	0.93 (2)	1.90 (2)	2.7943 (15)	161.7 (18)
O1—H1···N1	0.91 (2)	1.79 (2)	2.6913 (16)	168.7 (18)
